# Unilateral hyperpigmented lesion of the breast

**DOI:** 10.1002/ccr3.7132

**Published:** 2023-04-02

**Authors:** Kouki Chaima, Amouri Mariem, Sellami Khadija, Kallel Rim, Bahloul Emna, Boudawa Tahya, Turki Hamida

**Affiliations:** ^1^ Department of Dermatology Hospital of Hedi Chaker Sfax Tunisia; ^2^ Department of Anatomopathology Hospital of Habib Bourguiba Sfax Tunisia

**Keywords:** dermoscopy, hyperpigmenation, melanosis of the areola and nipple

## Abstract

Muco‐cutaneous melanosis is a benign entity with no progression. Although, dermoscopic features may help to differentiate melanosis from malignant pigmented diseases, histopathology remains crucial for the confirming of melanosis of the nipple and areola. Herein, we represent a new case of melanosis of the areola and we describe its clinico‐pathological aspects.

## 1 INTRODUCTION

Areola and nipple diseases are uncommon and remain under recognized. A cliniclo‐histopathology correlation is the key to achieve a correct diagnosis. Differential diagnoses of pigmented lesions of the nipple includes pigmented nipple melanocytic nevus, melanosis of the nipple, seborrheic keratosis, pigmented basal cell carcinoma, melanoma, and Paget disease.^1^ Melanosis of the areola and nipple (MAN) represents a new clinical entity. It is in the spectrum of benign melanosis, which includes melanosis of the vulva, penile lentigo, labial lentigo, and primary acquired melanosis of the conjunctiva.

We, herein, present a new case of MAN and describe its clinical, dermoscopic, and histological features.

## 2 CASE REPORT

**FIGURE 1:**
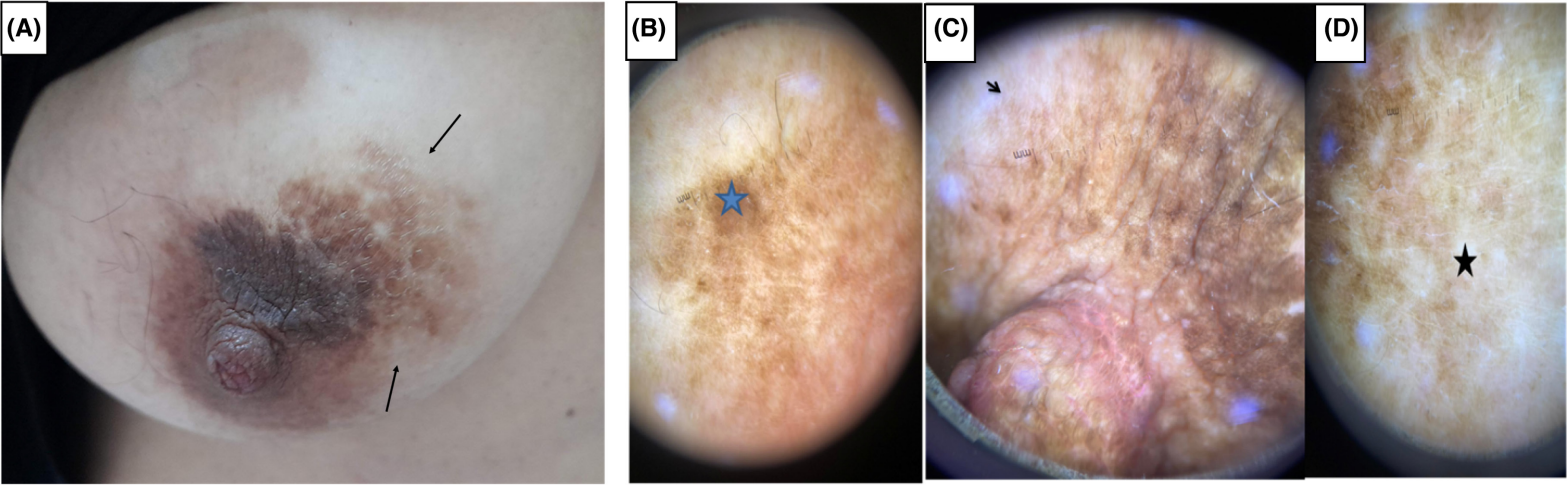
(A) A hyper‐pigmented macular plaque of 2.5 cm at the level of the right areola and overflowing on the skin of the breast with irregular borders. Non polarized dermoscopic features (B–D): White structureless areas (black asterix) and heterogeneous pigmented areas (orange asterix) with a reticular network at the periphery (black arrow).

**FIGURE 2:**
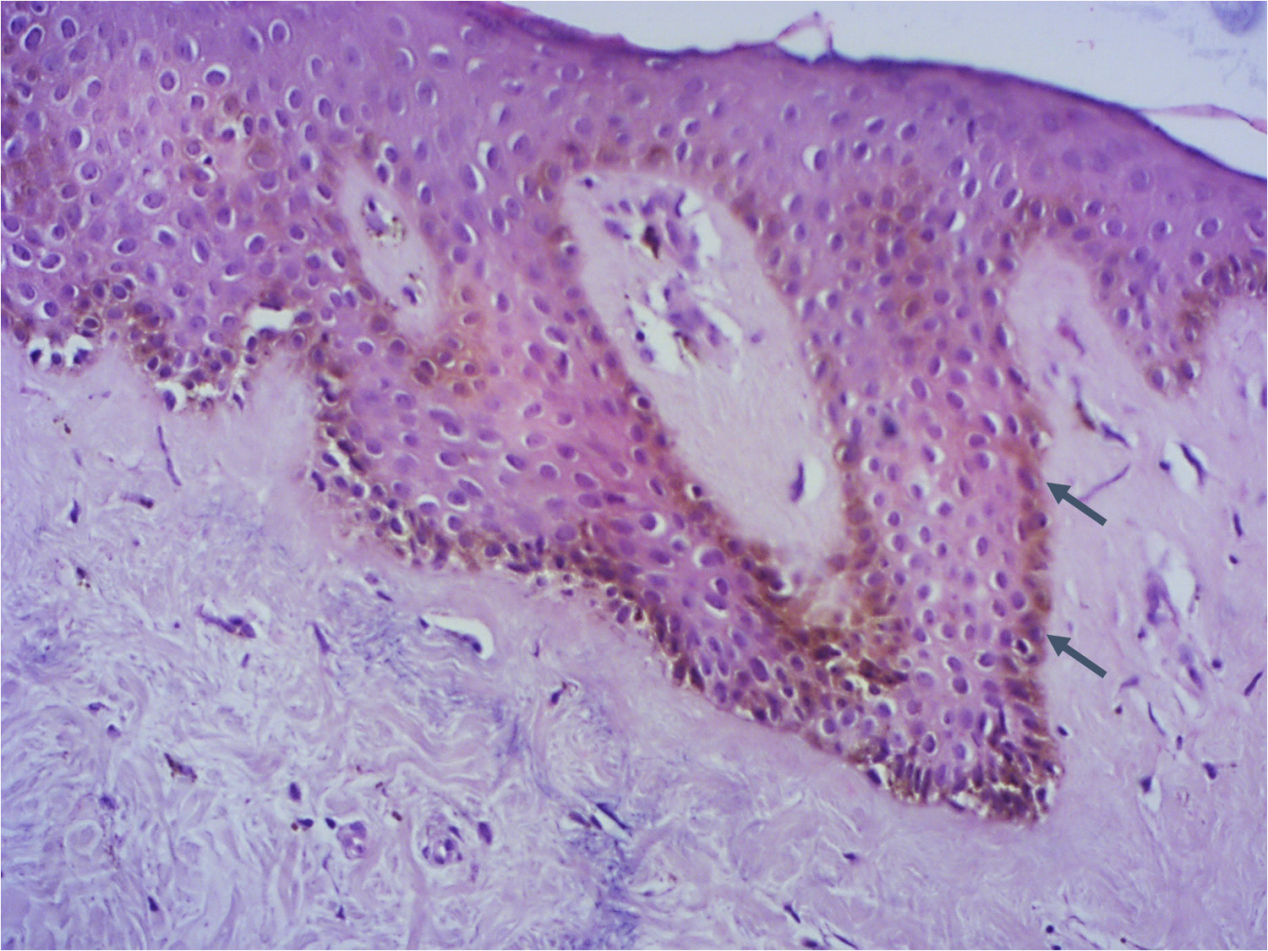
Acanthosis and hyperpigmentation of basal cells, especially at the rete pegs of the epidermis (HEx200).

A 35‐year‐old woman, with no significant pathological history presented with a pigmented patch of the right breast evolving for about 18 months. She denied any application of topical agents, wearing tight clothing or pre‐existing erythematosis lesion. The patient and her first‐degree relatives had no history of malignant tumors. She gave no history of any systemic illness or trauma. She had skin phototype IV. The clinical examination showed a rounded hyper‐pigmented macular plaque of 2.5 cm on the right areola that overflow on the surrounding skin with irregular borders (Figure 1A). On dermoscopy, we found white structureless areas and heterogeneous pigmented areas with a reticular network at the periphery (Figure 1B–D). A skin biopsy excluded cutaneous melanoma and pigmented Paget's disease, and showed epidermal acanthosis, hyperpigmentation of the basement membrane associated with numerous melanophages. There was neither cytonuclear atypia nor melanocytic hyperplasia (Figure 2). Immunostaining was negative for HMB‐45. The diagnosis of MAN was retained. We opted for abstention. Patient regular monitoring did not show any extension (within a 6‐month follow‐up).

## 3 DISCUSSION

MAN was first described by Pittis and Barber in 1990,^1^ yet it is less common than genital melanosis. A PubMed search found only 11 cases of MAM by the end of 2021.^2^, ^3^, ^4^ Women between 25 and 40 years old are most affected. Areola melanosis belongs to mucocutaneous melanosis (melanosis of lips, mouth, perianal, hands, and feet). The pathophysiology of this hyperpigmentation is unclear. The role of hormones remains unproven, even if most reported cases have occurred in pregnant women. In fact, hyperpigmentation is a well‐known pregnancy‐related change and is more marked in normally hyperpigmented areas, including the areola. Melanocytes at these sites may exhibit increased sensitivity to hormonal stimulation.^5^ Clinical features that can mimic melanoma, include asymmetry, irregular borders, color changes, and large size, may be of concern to patients and physicians. Given the rarity of this entity, dermoscopy patterns have not been fully established.^2^, ^3^ Most MAN cases show a cobblestone pattern, representing the texture of normal skin on the areola. Besides, annular cobblestone structures, lighter brown rings in the center, reticular structures, narrow parallel lines, and atypical pigmented networks represent other dermoscopic patterns^5^ In our patient, white unstructured and heterogeneously pigmented areas with surrounded by reticulated network were seen. Primary malignant melanoma of breast skin accounts for less than 5% of all malignant melanomas, and malignant melanoma of the nipple and areola constitutes about 12% of primary malignant melanomas of breast skin. The incidence of malignant melanoma of the breast is increasing and comprises other neoplasms.^6^ Malignant melanoma of the nipple and areola can be missed because of skin pigmentation and because the breast is usually covered by clothing. Melanoma, pigmented Paget disease, and melanopathy of the nipple and areola cannot be distinguished clinically from MAN. Therefore, histopathological confirmation is mandatory. We have described typical findings in our case of areola and nipple melanosis. These include acanthosis, basal hyperpigmentation, scattered melanocytes in the papillary dermis, and melanocytes with prominent dendritic protrusions. There are no features of melanoma, such as cytological atypia, pagetoid pattern, or melanocyte aggregate.^3^ Given to the rarity of reported pigmented lesions of the nipple and areola, dermoscopy patterns are not well established. Nina Kate et al. described the correlation between dermoscopy and histological findings. The atypical pigment network seen on dermoscopy corresponds to areas with increased epidermal pigment. Melanocytes in the dermis surrounding the ridge correspond to gray areas with a uniform appearance. No treatment is required. Follow‐up examination is recommended.^5^

In conclusion, melanosis is a benign entity with no progression. In that respect, no further intervention is required. Although, dermoscopic features may help to differentiate melanosis from malignant pigmented diseases, histopathology remains crucial for the confirming of MAN.

## AUTHOR CONTRIBUTIONS

**Kouki Chaima:** Conceptualization; data curation; supervision; validation; writing – original draft. **Amouri Mariem:** Conceptualization; data curation; visualization; writing – original draft. **Sellami Khadija:** Data curation; formal analysis; software; supervision. **Kallel Rim:** Conceptualization; funding acquisition; software; supervision; writing – original draft. **Bahloul Emna:** Conceptualization; visualization; writing – original draft. **Boudawara Tahya:** Conceptualization; visualization; writing – original draft. **Turki Hamida:** Visualization; writing – original draft; writing – review and editing.

## CONSENT

Written informed consent was obtained from the patient to publish this report in accordance with the journal's patient consent policy.

## Data Availability

None.

## References

[1] Pittis JD , Barber FA . Melanosis of the areola. Arch Dermatol. 1990;126(4):542‐543. 2322004 10.1001/archderm.126.4.542

[2] Blum A , Metzler G , Caroli U . Melanosis of the areola in dermoscopy. J Am Acad Dermatol. 2004;51:664‐665. 15455482 10.1016/j.jaad.2004.04.011

[3] Mikhail M , Sceppa J , Smith BL , Chu P , Marghoob AA . Four views of areolar melanosis: clinical appearance, dermoscopy, confocal microscopy, and histopathology. Dermatol Surg. 2008;34(8):1101‐1103. 18462414 10.1111/j.1524-4725.2008.34219.x

[4] Isbary G , Coras‐Stepanek B , Dyall‐Smith D , Guther S , Tillmann A , Stolz W . Five patients with melanosis of the nipple and areola clinically mimicking melanoma. J Eur Acad Dermatol Venereol. 2014;28(9):1251‐1254. 23919913 10.1111/jdv.12225

[5] Antonov NK , Bosenberg MW , Halasz CL . Melanosis of the areola and nipple with an atypical pigment network. Int J Dermatol. 2016;55(7):811‐813. 26220624 10.1111/ijd.12898

[6] Mahmoudzadeh L , Abbasi A , Mahmodlou R , Aghayan S , Asghari R . Malignant melanoma of the nipple with axillary metastasis. JCO Oncol Pract. 2020;16(8):516‐518. 32539653 10.1200/JOP.19.00803

